# Concurrent Physical Activity Protects Against C26 Adenocarcinoma Tumor-Mediated Cardiac and Skeletal Muscle Dysfunction and Wasting in Males

**DOI:** 10.3390/cells14120924

**Published:** 2025-06-18

**Authors:** Louisa Tichy, Kimberly F. Allred, Erika T. Rezeli, Michael F. Coleman, Clinton D. Allred, Stephen D. Hursting, Traci L. Parry

**Affiliations:** 1 Department of Kinesiology, University of North Carolina at Greensboro, Greensboro, NC 27412, USA; l_tichy@uncg.edu; 2 Department of Nutrition, University of North Carolina at Greensboro, Greensboro, NC 27412, USA; kfallred@uncg.edu (K.F.A.); cdallred@uncg.edu (C.D.A.); 3 Department of Nutrition, University of North Carolina at Chapel Hill, Chapel Hill, NC 27599, USA; tuerika@email.unc.edu (E.T.R.); mcoleman@unc.edu (M.F.C.); hursting@email.unc.edu (S.D.H.)

**Keywords:** cancer, muscle wasting, exercise, physical activity, protection

## Abstract

Muscle loss unresponsive to nutritional supplementation affects up to 80% of cancer patients and severely reduces survival and treatment response. Exercise may help preserve muscle mass and function, yet the translatability of preclinical methods remains questionable. This study aimed to assess how voluntary wheel running, a clinically relevant physical activity, protects skeletal and cardiac muscle against cancer-mediated dysfunction and identify underlying molecular mechanisms. Methods: BALB/c mice were assigned to sedentary nontumor-bearing (SED+NT), sedentary tumor-bearing (SED+T), wheel run nontumor-bearing (WR+NT), and wheel run tumor-bearing (WR+T). Tumor-bearing groups received 5 × 10^5^ C26 cells; WR mice had wheel access for 4 weeks. Muscle function and tissue were analyzed for protective mechanisms. Results: SED+T mice exhibited significant fat and lean mass loss, indicating cachexia, which was prevented in WR+T mice. SED+T also showed 15% reduced grip strength and cardiac dysfunction, while WR+T preserved function. WR+T mice had lower expression of muscle wasting markers (Atrogin1, MuRF1, GDF15, GDF8/11). Physical activity also reduced tumor mass by 57% and volume by 37%. Conclusion: Voluntary wheel running confers tumor-suppressive, myoprotective, and cardioprotective effects. These findings support physical activity as a non-pharmacological strategy to combat cancer-related muscle wasting and dysfunction.

## 1. Introduction

Cancer is a major health and economic challenge and remains one of the leading causes of death worldwide [1]. Up to 80% of cancer patients suffer from cancer-mediated muscle wasting, also known as cancer cachexia, resulting in skeletal and cardiac muscle dysfunction and atrophy [2]. An inverse relationship between skeletal muscle loss and overall chances of survival exists, suggesting the importance of muscle protection and preservation to attenuate cachexia, prolong survival, and improve quality of life in cancer patients [3].

To date, there are limited effective prevention and treatment options against cancer cachexia, which cannot be reversed by conventional nutritional support [4]. Many cachectic cancer patients progress quickly to advanced stages of cachexia and become unresponsive to traditional anticancer therapies [5]. The underlying cellular mechanisms of cancer cachexia, and specifically cancer-mediated skeletal and cardiac muscle wasting, remain not well understood. The main drivers of this disease seem to be an imbalance between protein synthesis and degradation, leading to disruption of protein homeostasis via an upregulation of systemic inflammation, abnormal metabolism, and upregulated degradation pathways [6,7,8]. However, as it is a multifactorial disease that heavily relies on multiorgan crosstalk, the contributions and regulations of each pathway are complex [9]. Future interventions and treatment options need to consider a multifactorial approach targeting a combination of dysfunctional pathways associated with cancer-mediated muscle wasting [10,11].

In the past decade, physical activity and exercise have received increased attention as a potential non-pharmacological, easily accessible, and affordable adjuvant to traditional anticancer therapies [12,13,14]. Encouragingly, investigation of the benefits of exercise in cancer survivors is growing, and from 2010 to 2018, the number of randomized controlled trials increased by 281% [13]. However, due to several practical and ethical constraints associated with physical activity and cachexia research in the clinical setting, most exercise oncology research with a focus on cancer cachexia has been conducted in the preclinical setting. In animal models of cachexia, forced treadmill exercise of moderate-to-high intensities specifically (or even low-intensity exercise) has been shown to protect skeletal and cardiac muscle from cancer-mediated dysfunction and atrophy, and inhibit tumor growth via multifactorial regulation of cellular mechanisms, such as inflammation, metabolism, and degradation pathways [15,16,17,18]. However, several factors hamper the translation of treadmill running in rodent studies to humans, including the forced nature of the intervention in a stressful environment, food/water inaccessibility during typically long bouts of exercise, the regular use of negative stimuli (e.g., electric shock), and species-specific physiological and anatomic differences in response to treadmill exercise [19]. Another mode of physical activity that offers advantages in utility and translatability of exercise interventions to the clinical setting is voluntary wheel running. In this setup, mice have unlimited access to running wheels in their individual cages, allowing mice to self-select timing and duration of exercise that closely mirrors their natural behavior in the clinical setting [19]. Therefore, this study aimed to identify if physical activity in the form of unlimited access to running wheels can protect skeletal and cardiac muscle against cancer-mediated muscle wasting and dysfunction.

## 2. Materials & Methods

### 2.1. Experimental Design

The study was performed on 8–10-week-old male BALB/c mice purchased from the Jackson Laboratory (JAX stock #000651). Throughout the study protocol, all mice were housed in standard mouse cages and provided with distilled water and standard rodent chow ad libitum in a temperature-controlled animal facility with a 12:12 light-dark cycle. All procedures were approved by the University of North Carolina at Greensboro’s Institutional Animal Care and Use Committee and complied with the Animal Welfare Act guidelines. Mice were randomly selected and separated into four groups: sedentary nontumor -bearing (SED+NT; *n* = 10), sedentary tumor-bearing (SED+T; *n* = 10), wheel-running nontumor-bearing (WR+NT; *n* = 10), wheel-running tumor-bearing (WR+T; *n* = 10). Sample size was chosen based on power calculations (power, 80%; alpha: 0.05). Animals were either inoculated with tumor cells (T groups; 5 × 10^5^ C26 cells in right flank) or were sham injected and remained tumor-free (NT groups) for 4 weeks. During the subsequent 4 weeks of tumor progression, mice either had unlimited access to running wheels (WR groups) or remained sedentary (SED groups). Grip strength was measured, echocardiography was performed to assess cardiac muscle structure and function, and tumor sizes were recorded using calipers throughout the study. All animals were sacrificed at the end of the 4-week study protocol. A subset of randomly selected mice (*n* = 5/group) were assessed for body composition (postmortem) by magnetic resonance (EchoMRI, Houston, TX, USA). Blood was collected via cardiac puncture, and complete blood counts were performed immediately. Gastrocnemius, heart, spleen, and tumor tissues were collected, weighed, and flash frozen in liquid nitrogen for subsequent biochemical analysis.

### 2.2. Tumor Model

The colon-26 (C26) model is a commonly used murine colon adenocarcinoma transplant model of cancer cachexia. C26 cells were generously provided by the Andrea Bonetto lab (University of Colorado School of Medicine) and were used to grow subcutaneous tumors in the right flank of T mice. Cells were grown in Dulbecco’s Modified Eagle Medium (DMEM; ATCC #30-2002; Manassas, VA, USA) in an incubator that was set to 5% CO_2_ and 37 °C; the medium was supplemented with 10% fetal bovine serum. On the day of the tumor injections, cells were treated with trypsin for cell dissociation, and viability counts were performed. On day 1, mice in T groups were inoculated with a concentration of 5 × 10^5^ C26 cells in 150 μL of sterile phosphate buffered saline (PBS; Gibco, Waltham, MA, USA) subcutaneously in the right flank, and mice in NT groups were given a sham injection of PBS of equivalent volume [20]. Body weight, body conditioning, and tumor measurements were taken three times each week. Tumor length, width, and thickness were measured three times per week using a Vernier caliper. Tumor measurements were used to calculate relative tumor mass and tumor volume using the following formulas, in which a equals the longest diameter and b equals the shortest diameter of the tumor [21]: (1) relative tumor mass = wet tumor mass/(total body mass-wet tumor mass); (2) tumor volume (mm^3^) = (a*b^2)/2. If estimated tumor mass exceeded 25% of body mass, percent loss of tumor free body mass exceeded 25% of starting mass, tumor became ulcerated, or the animal received a body conditioning score of less than 2, mice were to be euthanized and removed from the study [17]. One mouse in the SED+T group met endpoint criteria prior to the end of the 4-week study period and was removed from the study (therefore, SED+T: *n* = 9). Data of the present study reflects only animals that reached the end of the 4-week tumor bearing period.

### 2.3. Running Wheels

Mice were separated into wheel running (WR) and sedentary (SED) groups. Mice in WR groups were single-housed and had unlimited access to running wheels in their cages for 4 weeks. During those 4 weeks, SED mice did not have access to running wheels and remained sedentary. Throughout the study, running wheels were connected to a computer equipped with VitalView 6 software (Starr Life Sciences Corp., Oakmont, PA, USA) to record wheel running data as revolutions and distance in kilometers per hour. Total distance in kilometers was calculated at the endpoint of the study.

### 2.4. Grip Strength

Grip strength was assessed at baseline and sacrifice timepoints. Mice grasped a metal grid, attached to a force meter (Harvard Apparatus, Holliston, MA, USA), and front and back legs and were gently pulled horizontally in the opposite direction of the force meter 3–5 times per test [22]. Peak force produced was measured in grams and the average of all three trials was normalized to body mass. Due to the noninvasive nature of this test and the proneness of mice to becoming habituated and losing interest in the task, mice were not allowed trial runs prior to data collection.

### 2.5. Echocardiography

At baseline and sacrifice timepoints, mice were loosely restrained in a supine position. Hair was removed from the chest by means of a depilatory agent. Transduction jelly was applied to the chest and two-dimensional M-mode echocardiography (GE Vivid 7 Dimension) was performed in the parasternal long-axis view at the level of the papillary muscle. Measurements represent an average of three cardiac cycles from each animal. Anterior and posterior wall thickness was measured as distance from endocardial to epicardial leading edges. Left ventricular (LV) diameter was measured and cardiac function was assessed by fractional shortening: (FS)% = [(LV end diastolic diameter − LV end systolic diameter)/LV end systolic diameter] * 100.

### 2.6. Sacrifice and Harvest

All mice were sacrificed at the end of the 4-week protocol via isoflurane anesthesia, followed by cervical dislocation. Prior to cervical dislocation, blood was collected via cardiac puncture and Complete Blood Counts (CBC) were immediately evaluated using an Abaxis VetScan HM5C Hematology Analyzer (Zoetis; Parsippany, NJ, USA). Gastrocnemius and cardiac muscle tissue were collected into 1.5 mL cryogenic storage vials and immediately flash frozen in liquid nitrogen. All tissues were stored at −80 °C until further analysis.

### 2.7. Protein Expression

Approximately, 25–45 mg of cardiac and gastrocnemius muscle tissue was homogenized via 8M Urea lysis buffer with a Qiagen TissueLyser LT homogenizer (Hilden, Germany). Homogenates were centrifuged for 10 min at 12,500× *g* at 4 °C, and the supernatant was collected for protein analysis. Protein concentration was determined via Bradford method [23]. A total of 28 μg of protein in cardiac tissue and 47 μg of protein in gastrocnemius tissue per sample was loaded onto 4–12% Bis-Tris gels and separated by NuPAGE MES running buffer (Thermofisher, MA, USA) for 30–40 min at constant 200 V. Proteins were then transferred to PVDF membranes for 60 min at constant 30 V. PVDF membranes were blocked in 5% nonfat milk powder in TBST for an hour at room temperature, and then incubated with primary antibody diluted in 5% milk powder in TBST (anti-Atrogin1, 1:500 dilution [SC-166806]; anti-MuRF1, 1:500 dilution [SC-398608]; anti-GDF15, 1:500 dilution [SC-515675]; anti-GDF8/11, 1:500 dilution [SC-398333]; anti-Beclin1, 1:1000 dilution [CST3495]; anti-IFNɣ; 1:500 dilution [CST8455]; and anti-P-STAT3; 1:500 dilution [CST9145]) at 4 °C overnight. Membranes were then incubated with species-specific secondary antibodies diluted in 5% milk powder in TBST (rabbit, 1:5000 [Sigma A9169; Sigma-Aldrich; St. Louisa, MO, USA]; mouse, 1:10,000 [Sigma A2228; Sigma-Aldrich; St. Louisa, MO, USA]) for one hour at room temperature. ECL select (GE Healthcare, RPN2235; GE HealthCare Technologies; Chicago, IL, USA) was used to develop membranes, which were imaged using a Bio-Rad Chem-Doc XRS+ (Hercules, CA, USA). Band intensity was quantified using Quantity One (Bio Rad; Hercules, CA, USA). Membranes were probed for anti-GAPDH (1:4000 [Sigma 039M4772V; Sigma-Aldrich; St. Louisa, MO, USA] in 5% milk powder in TBST) as a loading control protein overnight at 4 °C, and then incubated with species-specific secondary antibody (mouse; 1:10,000, diluted in 5% milk powder in TBST) for one hour at room temperature. Membranes were developed in ECL, imaged, and band intensity was quantified using Quantity One. Densitometry (Bio-Rad, Hercules, CA, USA) was used to determine protein concentrations. Concentrations of primary protein detection was normalized to the loading control GAPDH.

### 2.8. Statistical Analysis

All data are presented as mean ± standard deviation (SD). The statistical software GraphPad Prism (Version 10; La Jolla, CA, USA) was used to perform all statistics. Two-way ANOVA was performed to determine differences effects of activity protocol and tumor burden for each variable between groups. Two-way repeated measures ANOVA was performed to compare baseline and sacrifice measurements of select variables. Change score measurements were performed to calculate percent changes from baseline to sacrifice for select variables. Tumor characteristic differences were analyzed via Student’s *t*-test. All analyses were two-tailed and an alpha level of 0.05 was used to define statistical significance. If a significant difference (*p* < 0.05) was identified for ANOVA testing, Tukey’s post hoc testing was performed to identify where the significant differences occurred.

## 3. Results

### 3.1. Voluntary Physical Activity Is Well-Tolerated by Tumor-Bearing Mice

**Figure 1:**
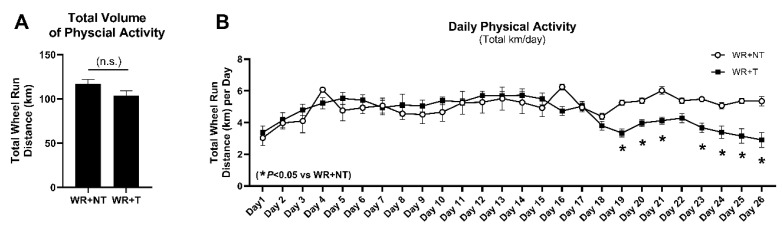
Comparison of total physical activity volume (**A**) and daily physical activity throughout the 4-week study (**B**) between WR+NT and WR+T groups. Physical activity was measured as distance in kilometers run on a mouse wheel. WR+NT, wheel running non-tumor bearing (*n* = 10); WR+T, wheel running tumor bearing (*n* = 10). *N* = 20. Values are reported as Mean ± SD. Significant difference: * *p* < 0.05, n.s., no significant difference.

Starting on day 1 of the study protocol, mice in WR groups had unlimited access to running wheels in their individual cages for 4 weeks. Revolutions per hour, kilometers per hour, and total distance run were measured via VitalView software. Total volume of physical activity (Figure 1A) and daily physical activity in kilometers per day (Figure 1B) were analyzed and compared between WR+T and WR+NT controls. Physical activity levels (km/day, Figure 1B) increased in both groups throughout the first week due to acclimation and familiarization with the wheels. Once tumors reached roughly 1cm in width (~day 14), physical activity (kilometers run per day) of mice in the WR+T group steadily declined until the end of the 4-week study (Figure 1B). While overall total volume of physical activity (Figure 1A) was not significantly different between groups, daily physical activity in the WR+T group was significantly lower starting on day 19 when compared to NT counterparts (*p* < 0.05; Figure 1B). However, despite the decline in daily running distance, a threshold of physical activity was maintained in both groups, regardless of tumor-bearing state, until the end of the protocol. Therefore, even though the WR+T mice ran less compared to WR+NT controls at the end of the study, WR+T mice still maintained a level of physical activity that provided protective effects against tumor-mediated muscle dysfunction and atrophy. These data indicate that physical activity was still well tolerated in the tumor-bearing group and supports the viability of physical activity and exercise as an adjuvant anticancer and anti-muscle wasting therapy.

### 3.2. Physical Activity Protects Against C26 Tumor-Mediated Body Wasting

**Figure 2:**
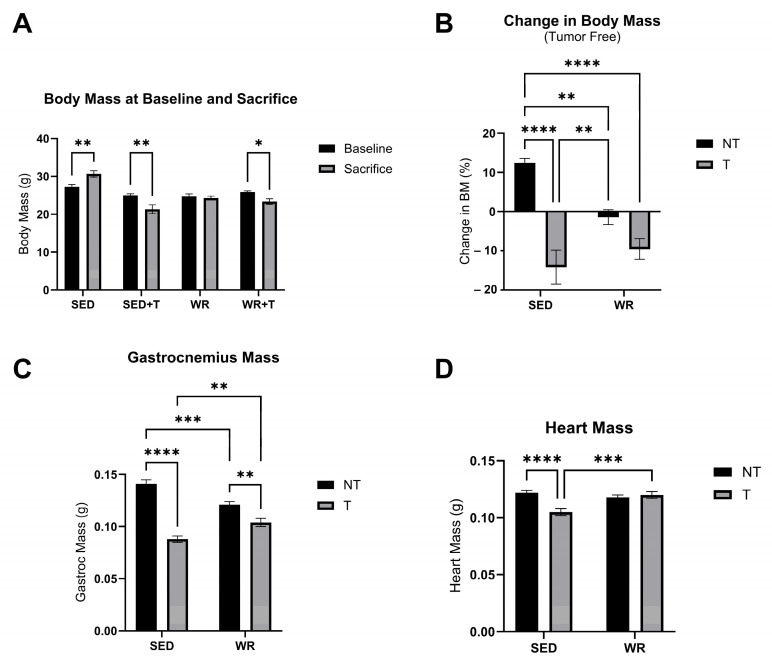
Comparison of baseline and sacrifice body mass (**A**), change score measurements (%) of body mass over time (**B**), gastrocnemius (**C**) and heart (**D**) muscle mass. Change scores were calculated by subtracting baseline from sacrifice scores and dividing results by baseline scores. Body mass is reported as tumor free (tumor mass is subtracted from overall body mass). SED, sedentary non-tumor bearing (*n* = 10); SED+T, sedentary tumor bearing (*n* = 9); WR, wheel running non-tumor bearing (*n* = 10); WR+T, wheel running tumor bearing (*n* = 10). *N* = 39. Values are reported as Mean ± SD. Significant difference: * *p* < 0.05, ** *p* < 0.01, *** *p* < 0.001, **** *p* < 0.0001.

**Figure 3:**
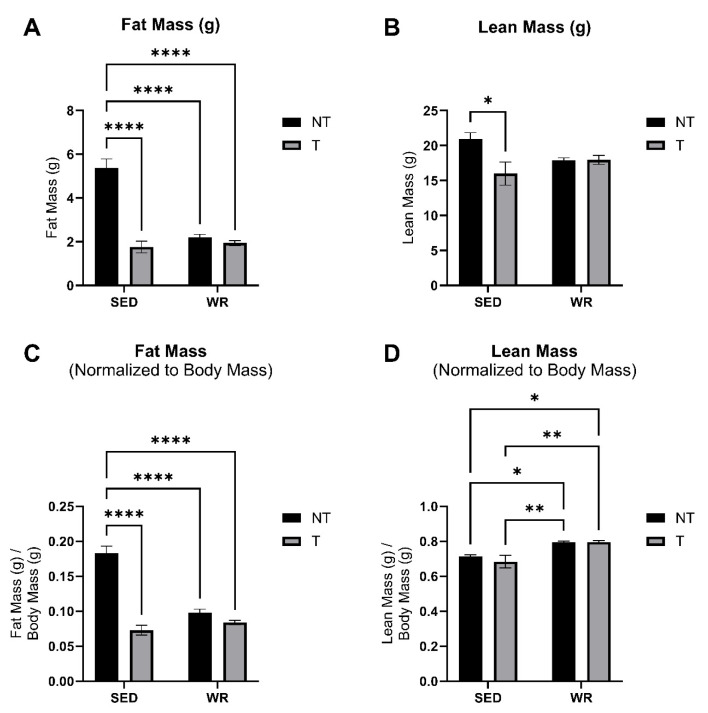
Comparisons of absolute fat mass (**A**), absolute lean mass (**B**), fat mass relative to body weight (**C**), and lean mass relative to body weight (**D**). SED+NT, sedentary non-tumor bearing (*n* = 10); SED+T, sedentary tumor bearing (*n* = 9); WR+NT, wheel running non-tumor bearing (*n* = 10); WR+T, wheel running tumor bearing (*n* = 10). *N* = 39. Values are reported as Mean ± SD. Significant difference: * *p* < 0.05, ** *p* < 0.01, **** *p* < 0.0001.

Body mass was measured at baseline and sacrifice timepoints. Cardiac and gastrocnemius skeletal muscle tissue were collected and weighed at the endpoint of the study. Body condition (fat mass and lean mass) was also measured at the endpoint of the study. A two-way repeated measures ANOVA revealed that SED+T and WR+T groups experienced significant body mass loss from baseline to sacrifice (Figure 2A; *p* < 0.05). However, the SED+T group experienced the greatest overall loss in body mass over time (−11%; *p* < 0.05; Figure 2B). Similarly, at the end of the study, fat mass (Figure 3A,C) and lean mass (Figure 3B,D) were lowest in the SED+T group compared to all other groups, and significantly lower than the SED+NT control group (*p* < 0.05), indicating progressive cancer cachexia. The SED+T group also had the lowest mixed fiber gastrocnemius skeletal muscle mass and heart mass compared to all other groups (Figure 2C,D). Collectively, this data indicates that our preclinical male colorectal cancer (C26) model elicited significant declines in lean mass and fat mass, resulting in a severe wasting phenotype.

WR appears to provide a protective effect in maintaining both lean and fat mass. In our male C26 mouse model, WR+T mice were not significantly different from SED+NT in terms of change in body mass over the course of the study (Figure 2A), lean mass (Figure 3B), skeletal muscle mass (Figure 2C), and heart mass (Figure 2D). In fact, when comparing WR+T vs. SED+T, our physical activity intervention resulted in significant benefits, including preservation of skeletal muscle mass (Figure 2C), heart mass (Figure 2D), and lean mass (Figure 3D). Even though the WR+T group ran less per day compared to WR+NT controls in the final ~10 days of the study, there still appears to be a training effect on lean mass via voluntary wheel running (WR). In particular, the WR groups have significantly greater lean mass compared to SED groups (Figure 3D). Similarly, WR training protected the heart from tumor-mediated cardiac atrophy (Figure 2D). These data indicate that even small amounts of physical activity during tumor bearing can play an important role in protecting the skeletal and heart muscle from tumor-mediated muscle wasting.

### 3.3. Voluntary Wheel Running Stunts Tumor Growth

**Figure 4:**
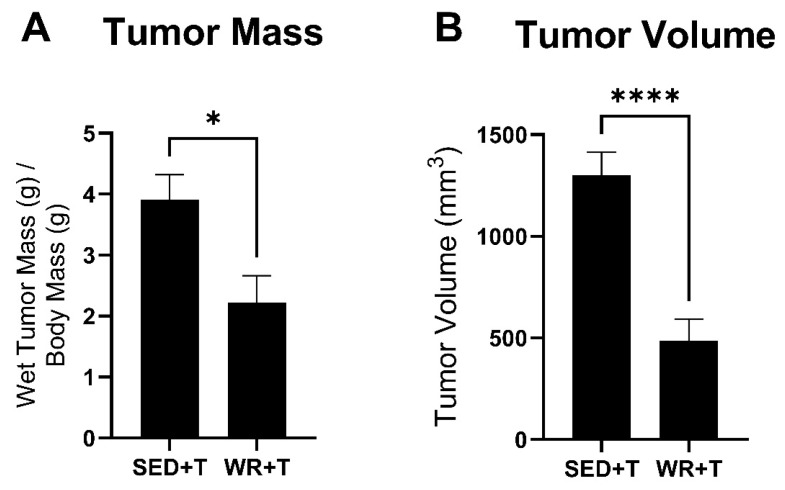
Tumor characteristics of SED+T and WR+T mice. Wet tumor mass was normalized to body mass (**A**). Estimated tumor volume (**B**) was calculated by using the following equation: tumor volume (mm^3^) = (a*b^2)/2, with a being the longest and b being the shortest diameter of the tumor. SED+T, sedentary tumor bearing (*n* = 9); WR+T, wheel running tumor bearing (*n* = 10). *N* = 19. Values are reported as Mean ± SD. Significant difference: * *p* < 0.05, **** *p* < 0.0001.

Tumor growth assessments were performed three times each week. Width, length, and thickness of tumors were measured and recorded once visible and palpable tumors were detected. Analysis of final tumor growth assessments determined that physically active mice (WR+T group) exhibited significantly smaller tumors based on relative tumor mass (Figure 4A) and tumor volume (Figure 4B) compared to sedentary counterparts (*p* < 0.05). Tumor evaluation showed a 57% decrease in wet tumor mass (Figure 4A) and a 37% decrease in estimated tumor volume (Figure 4B) in the physically active WR+T group compared to tumors in sedentary mice. These data indicated that, while physical activity levels decreased once tumors were palpable, mice in the WR+T group still experienced exercise-mediated anti-tumor and protective effects. The observed exercise-mediated stunting of tumor growth may in part provide exercise-mediated protection at the muscle level.

### 3.4. Tumor Bearing Influences White Blood Cell Profile

**Figure 5:**
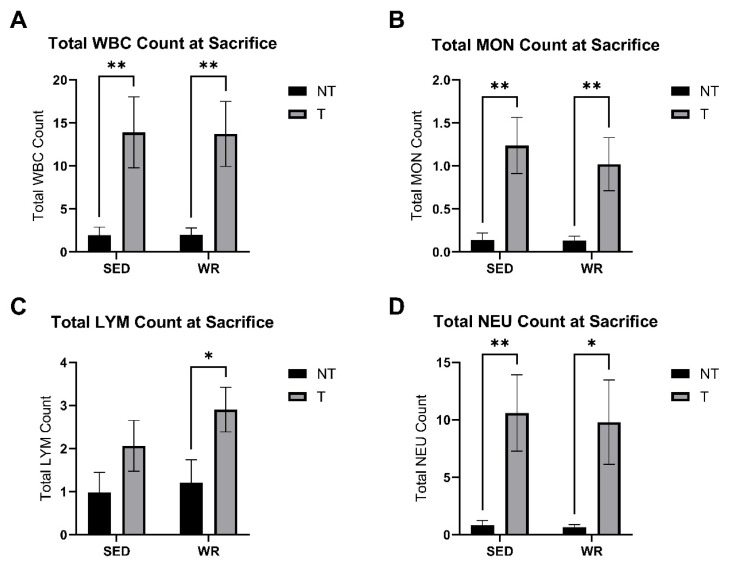
Comparisons of total circulating white blood cell counts (**A**), circulating monocyte counts (**B**), circulating lymphocyte counts (**C**) and circulating neutrophil counts (**D**). Blood was collected via cardiac puncture and complete blood counts were performed immediately to assess circulating immune cell counts. SED+NT, sedentary non-tumor bearing (*n* = 10); SED+T, sedentary tumor bearing (*n* = 9); WR+NT, wheel running non-tumor bearing (*n* = 10); WR+T, wheel running tumor bearing (*n* = 10). *N* = 39. Values are reported as Mean ± SD. Significant difference: * *p* < 0.05, ** *p* < 0.01.

Blood was collected via cardiac puncture, and complete blood counts were performed immediately to assess circulating immune cell counts. Two-way ANOVA was performed to analyze the effects of activity level (SED vs. WR) and tumor burden (NT vs. T) on circulating white blood cell counts (i.e., WBC, LYM, NEU, MON). Two-way ANOVA revealed that there was not a statistically significant interaction between the effects of tumor burden and activity level. Simple main effects analysis showed that tumor burden did have a statistically significant effect (*p* < 0.05) on total WBC, MON, LYM, and NEU counts at sacrifice (Figure 5A–D). SED+T and WR+T experienced significantly more white blood cell counts at sacrifice blood collection compared to NT counterparts (*p* < 0.05; Figure 5). The data suggest that tumor burden alone increases circulating white blood cell counts that are not attenuated by physical activity.

### 3.5. Physical Activity Protects Against C26 Tumor-Mediated Cardiac Dysfunction

**Figure 6:**
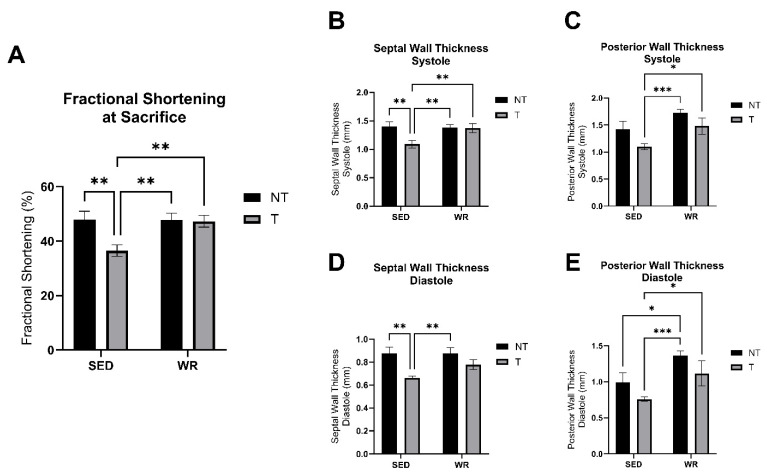
Cardiac function and cardiac geometry measures at systole and diastole. Fractional shortening as a measure of cardiac function (**A**) and septal and posterior wall thicknesses during systole and diastole (**B**–**E**) represents an average of three consecutive cardiac cycles per mouse measured via echocardiography. SED+NT, sedentary non-tumor bearing (*n* = 10); SED+T, sedentary tumor bearing (*n* = 9); WR+NT, wheel running non-tumor bearing (*n* = 10); WR+T, wheel running tumor bearing (*n* = 10). *N* = 39. Values are reported as Mean ± SD. Significant difference: * *p* < 0.05, ** *p* < 0.01, *** *p* < 0.001.

Cardiac structure and functional changes were tracked throughout the study via conscious echocardiography. No differences in cardiac function, measured as fractional shortening, were detected between groups at the baseline timepoint. At the endpoint of the study, SED+T mice experienced abnormalities in cardiac geometry. Two-way ANOVA, analyzing the effect of physical activity (SED vs. WR) and tumor burden (NT vs. T), revealed that septal wall thicknesses at systole and diastole were significantly thinner in the SED+T group compared to SED+NT mice (*p* < 0.05; Figure 6B,D), and posterior wall thicknesses at systole and diastole were significantly thinner in the SED+T group compared to WR+T counterparts (*p* < 0.05; Figure 6C,E). Additionally, fractional shortening, as a measure of cardiac function, was significantly declined in the SED+T group compared to all other groups (Figure 6A). Therefore, the data indicate that SED+T mice showed signs of tumor-mediated dilated cardiomyopathy with reduced fractional shortening. Tumor-mediated cardiac structural and functional changes were attenuated by physical activity as the WR+T did not show significant changes in cardiac geometry (Figure 6B–E) compared to NT groups and experienced preserved cardiac function (Figure 6A) compared to SED+T mice. Therefore, these data suggest that even small amounts of concurrent physical activity as an adjuvant anticancer treatment are beneficial in protecting cardiac structure and function from tumor-mediated dysfunctional changes.

**Figure 7:**
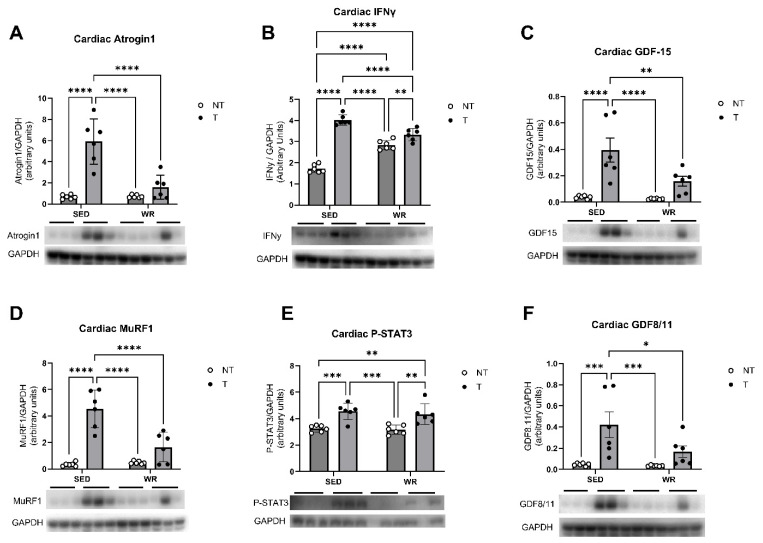
Effects of physical activity and tumor intervention on cardiac protein levels of Atrogin1 (**A**), IFNɣ (**B**), GDF-15 (**C**), MuRF1 (**D**), P-STAT3 (**E**), and GDF8/11 (**F**). SED+NT, sedentary non-tumor bearing (*n* = 6); SED+T, sedentary tumor bearing (*n* = 6); WR+NT, wheel running non-tumor bearing (*n* = 6); and WR+T, wheel running tumor bearing (*n* = 6). *N* = 24. Values are reported as Mean ± SD. Significant difference: * *p* < 0.05, ** *p* < 0.01, *** *p* < 0.001, **** *p* < 0.0001.

Analysis of cellular protein expression and molecular signaling pathways in cardiac tissue via western blotting sought to determine if the observed tumor-mediated cardiac dysfunction is attributable to enhanced muscle wasting and atrophy pathways, metabolic dysfunction, and/or inflammation. Cardiac tissue was analyzed for expression of muscle wasting-associated proteins MuRF1 (Figure 7D) and Atrogin1 (Figure 7A), GDF 15 and GDF8/11 (Figure 7C,F), and proteins associated with dysfunctional metabolism and inflammatory proteins IFNy and P-STAT3 (Figure 7B,E). Findings show significant cardioprotective effects of physical activity. Two-way ANOVA analysis revealed that expression of all analyzed proteins was significantly elevated in the SED+T group (*p* < 0.05; Figure 7) compared to SED+NT controls and WR+T mice, indicating that our sedentary C26 tumor model elicited increased muscle wasting, metabolic dysfunction, and inflammation. While WR+T mice experienced increased expression of P-STAT3 and IFNy compared to WR+NT counterparts, these protein expressions were lower compared to SED+T mice, indicating that while inflammation seems to be upregulated with tumor-bearing, physical activity has cardioprotective effects. These findings are in line with the cardiac structural and functional data described above, suggesting that physical activity, even at low levels, significantly protects the heart against tumor-mediated cardiac dysfunction and preserves cardiac structure. This may be attributed to physical activity’s ability to positively regulate cardiac muscle wasting, metabolic, and inflammatory pathways.

### 3.6. Physical Activity Protects Against C26 Tumor-Mediated Loss in Skeletal Muscle Strength

**Figure 8:**
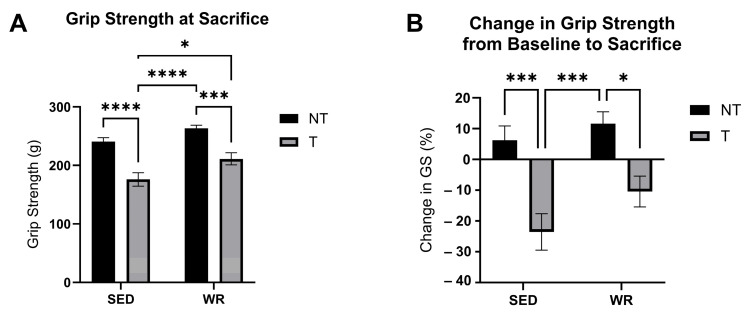
Comparisons of grip strength at the endpoint of the 4-week study (**A**) and chance scores (%) in grip strength from baseline to sacrifice (**B**). Grip strength represents an average of three grip strength trials per mouse measured via a force meter. Change scores were calculated by subtracting baseline from sacrifice scores and dividing results by baseline scores. SED+NT, sedentary non-tumor bearing (*n* = 10); SED+T, sedentary tumor bearing (*n* = 9); WR+NT, wheel running non-tumor bearing (*n* = 10); and WR+T, wheel running tumor bearing (*n* = 10). *N* = 39. Values are reported as Mean ± SD. Significant difference: * *p* < 0.05, *** *p* < 0.001, **** *p* < 0.0001.

Skeletal muscle function was tracked throughout the study via grip strength assessment. No differences in grip strength between groups was observed at baseline. By the end of the 4-week tumor-bearing study, SED+T mice experienced a negative change (−25%) in grip strength (Figure 8B) and showed significantly lower grip strength at the endpoint of the study compared to all other groups (*p* < 0.05; Figure 8A), whereas WR+T did not experience significantly different grip strength compared to sedentary controls (Figure 8A,B). In fact, the WR+T group exhibited significantly greater grip strength than SED+T mice at the end of the study (*p* < 0.05; Figure 8A). These data indicate that while being tumor-bearing for 4 weeks is associated with significant reductions in grip strength, even small amounts of physical activity show significant myoprotective effects. While physical activity levels declined throughout the tumor-bearing period, WR+T still showed preservation of muscle strength and function, indicating the therapeutic potential of low levels of physical activity to rescue skeletal muscle from tumor-mediated dysfunction.

**Figure 9:**
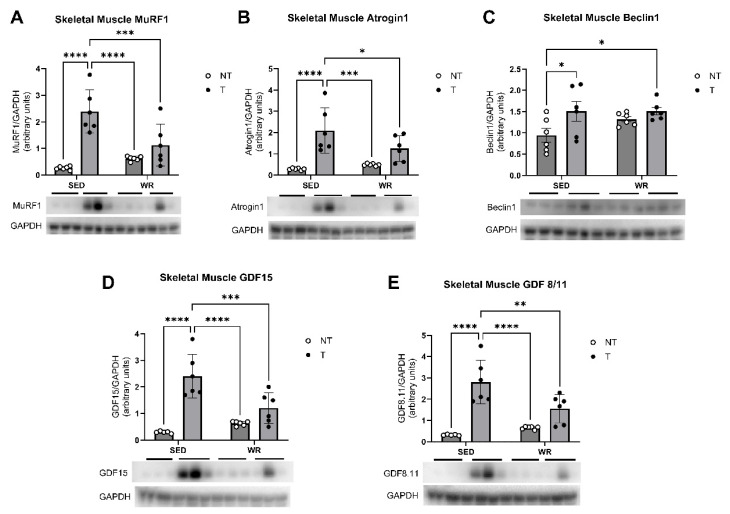
Effects of physical activity and tumor intervention on skeletal muscle protein levels of MuRF1 (**A**), Atrogin1 (**B**), Beclin1 (**C**), GDF-15 (**D**), and GDF8/11 (**E**). SED+NT, sedentary non-tumor bearing (*n* = 6); SED+T, sedentary tumor bearing (*n* = 6); WR+NT, wheel running non-tumor bearing (*n* = 6); and WR+T, wheel running tumor bearing (*n* = 6). *N* = 24. Values are reported as Mean ± SD. Significant difference: * *p* < 0.05, ** *p* < 0.01, *** *p* < 0.001, **** *p* < 0.0001.

Protein expression of muscle-wasting and atrophy pathways, metabolic dysfunction, and autophagy were assessed in gastrocnemius muscle tissue to determine if the observed differences in grip strength are attributable to changes in these signaling pathways. Two-way ANOVA findings of Western blot analyses indicated significant upregulation of autophagic protein Beclin1 expression in both tumor bearing groups compared to NT controls (*p* < 0.05; Figure 9C). Analyses also showed that muscle wasting-associated protein expression of MuRF1 and Atrogin1 (Figure 9A,B) and dysfunctional metabolism and inflammation-associated protein expression of GDF15 and GDF8/11 (Figure 9D,E) were increased in the SED+T group compared to all other groups. Two-way ANOVA revealed that physical activity seemed to protect against dysfunctional changes in muscle wasting, metabolism, and inflammatory signaling pathways as WR+T mice did not show significant differences in these protein expressions compared to NT controls. These data suggest that exercise-mediated protection of skeletal muscle via grip strength preservation coincided with lower expression of muscle wasting- and dysfunctional metabolism-associated proteins Atrogin1, MuRF1, GDF15, and GDF8/11 in WR+T compared to SED+T mice.

## 4. Discussion

The physiological benefits of exercise are well-established, not only for overall health but also as an adjuvant treatment for metabolic diseases, such as cardiovascular disease, obesity, and diabetes [24,25,26]. More recently, evidence from preclinical and clinical research has shown the potential of physical activity and exercise as an adjuvant anticancer therapy. Increased levels of physical activity are not only associated with a reduction in cancer risk, but also with increased survival rates, decreased tumor burden in preclinical models, and overall increased quality of life of cancer patients [12,13,14,27]. While research in exercise oncology is expanding, exercise as a protective measure against cancer-mediated muscle wasting remain understudied. Up to 80% of cancer patients suffer from cancer-mediated skeletal and cardiac muscle wasting, resulting in death of up to one-third of these patients [28]. Thus, additional studies on the effects, underlying mechanisms, and most beneficial modes of exercise in cancer cachexia are urgently needed.

Our study sought to determine if physical activity, in the form of concurrent voluntary wheel running, could protect skeletal and cardiac muscle against cancer-mediated muscle wasting and dysfunction in a cancer cachexia mouse model. While most preclinical exercise oncology studies have focused on forced treadmill running, mainly using high intensity exercise protocols [15,29,30], mice in our study had unlimited access to running wheels and were able to self-select their voluntary physical activity levels. Physical activity via voluntary wheel running mimics the circumstances seen in clinical settings better than preclinical forced treadmill exercise [31]. In the clinical setting, cancer patients will self-select their daily activity levels and, due to their chronically ill status, are likely to experience declines in self-selected physical activity levels and intensity [32]. In healthy mice, voluntary wheel running has been shown to induce skeletal and cardiac muscle adaptations that are similar to adaptions in endurance exercise [33]. Therefore, by allowing mice in our preclinical model to self-select their physical activity levels, our study mimics a real-world intervention for the management of cancer-mediated muscle wasting and dysfunction [16].

Similar to clinical observations [32], tumor-bearing mice in our study experienced a significant decline in daily physical activity levels compared to non-tumor controls, starting at approximately the same time as when tumors became palpable and visible. However, overall daily physical activity of even small amounts still showed significant protection of skeletal and cardiac muscle in WR+T compared to SED+T counterparts. In line with previous research focusing on the effects of moderate-to-high intensity forced treadmill exercise [15,18,34], even small amounts of physical activity resulted in significant grip strength and body mass preservation in WR+T compared to SED+T mice in our study. Additionally, physically active mice showed significantly smaller tumor sizes based on mass and volume, in line with previous preclinical studies indicating that exercise can stunt tumor growth [35,36,37]. Underlying mechanisms by which exercise can protect skeletal muscle and stunt tumor growth have been proposed to involve regulation of inflammatory and metabolic signaling pathways [38,39]. Therefore, it is possible that our concurrent physical activity intervention regulated inflammatory and metabolic signaling to stunt tumor growth while also eliciting myoprotective effects.

Our study has found significant upregulation in muscle wasting pathways in SED+T mice via increased GDF15, Atrogin1, and MuRF1 protein levels. Atrogin1 and MuRF1 are two E3 ubiquitin ligases that have been identified as key players in muscle loss and are highly expressed in skeletal muscle during muscle atrophy [40,41]. During cancer, these muscle-specific E3 ubiquitin ligases contribute to cancer-mediated muscle loss, one of the primary characteristics of cancer cachexia. In agreement with data from our study, preclinical cancer cachexia studies consistently show significant overexpression of Atrogin1 and MuRF1 in skeletal muscle tissue of cachectic animals and C2C12 immortalized mouse myoblast cells treated in vitro with TNFα [40,42]. In cancer patients with malignant disease, skeletal muscle biopsies expressed increased Atrogin1 and MuRF1 levels even prior to weight and muscle loss [42], suggesting potential of Atrogin1 and MuRF1 as clinical biomarkers of cancer-mediated muscle wasting. Targeting Atrogin1 and MuRF1 in knockout models or modulating these ubiquitin ligases through physical activity and exercise has been shown to have the ability to protect against and/or reverse muscle atrophy in patients suffering from sarcopenia [41].

Our study also identified protective effects of self-selected voluntary physical activity against muscle wasting in cachectic mice via modulation of Atrogin1 and MuRF1 signaling pathways—even with low volumes of physical activity. Modulation of these muscle wasting-associated signaling pathways likely play a role in the observed preservation of skeletal muscle function in vivo. Therefore, in line with previous sarcopenia research, our model suggests that physical activity has protective properties against muscle loss associated with cancer cachexia. Furthermore, modulation of MuRF1 may also assist with impeding tumor growth since MuRF1 knockout mice exhibit delayed pancreatic cancer growth [43]. Though not fully elucidated, targeting Atrogin1 and MuRF1 via physical activity or pharmacological inhibition holds value for successful anti-muscle wasting and anti-tumor growth therapy. Additionally, exercise has been shown to enhance the efficacy of anticancer drugs [44] while delaying disease progression, reducing cancer treatment toxic side effects, and improving cancer treatment completion rates [45]. Therefore, exercise may be a powerful adjuvant therapy to control both tumor growth and muscle wasting to ultimately improve survival rates and enhance quality of life of cancer survivors.

While the protective effects of exercise against skeletal muscle wasting and dysfunction have been studied previously, less information is known regarding the protective effects of voluntary wheel running against cancer-mediated cardiac dysfunction and remodeling. In vivo echocardiography has shown that tumor burden is able to induce cardiac remodeling and dysfunction, accompanied by abnormal cardiac metabolism and increased systemic inflammation in preclinical cachexia models [17,46,47]. However, the underlying pathways involved in these observations remain poorly understood. Our findings agree with previous studies. Additionally, our study explored multiple different signaling pathways and identified the primary involvement of muscle wasting and abnormal metabolism via upregulated expression of Atrogin1, MuRF1, GDF8/11, and GDF15 proteins in cardiac tissue. While the role of MuRF1 and Atrogin1 in muscle atrophy is better understood in skeletal muscle, these two muscle-specific E3 ubiquitin ligases also negatively regulate cardiac atrophy and dysfunction [48,49,50]. In cardiac patients and preclinical cardiomyopathy and heart failure models, Atrogin1 and MuRF1 promote the progression of cardiomyopathy and premature death due to cardiac dysfunction and remodeling [48,50]. In line with these findings in cardiomyopathy, our model suggests that Atrogin1 and MuRF1 are involved in cardiac remodeling and dysfunction associated with cancer and have a role in cancer cachexia. Similar to findings in skeletal muscle, cardiac muscle tissue can benefit from the protective properties associated with physical activity and exercise. While the anti-inflammatory and anabolic effects of exercise are well described in the literature [38,39], the effects of exercise and physical activity on Atrogin1 and MuRF1 expression and cancer-mediated cardiac cachexia remain unknown. Our study determined that even small amounts of physical activity in the form of voluntary wheel running induced cardioprotective effects by downregulating muscle wasting-associated signaling of Atrogin1 and MuRF1, in addition to regulating metabolic and pro-inflammatory pathways. Regulation of these catabolic pathways likely explain the preservation of cardiac structure and function observed in vivo in WR+T mice compared to SED+T counterparts.

One of the most novel findings of this study is the involvement of the muscle wasting marker GDF15 and GDF8/11 in the effects of exercise on skeletal and cardiac muscle wasting and dysfunction. GDF15 has recently been identified as a potential clinical biomarker to detect the progression and development of cachexia in cancer patients [51,52,53,54,55,56]. Independently of food intake, previous preclinical and clinical studies have suggested that GDF15 may contribute to muscle atrophy in cancer cachexia by interacting with the muscle-specific E3 ubiquitin ligases MuRF1 and Atrogin1 [57]. When treated with anti-GD15 antibody, skeletal muscle and body mass were preserved in a cachexia mouse model [58]. In cardiac muscle, increased circulating GDF11 levels have been associated with cardiac atrophy and cachexia [59]. However, despite a growing body of research on the role of GDF15 and GDF11 in skeletal and cardiac muscle loss in cancer cachexia, the underlying mechanisms by which these growth differentiation factors facilitate muscle loss remain unknown. Findings of our study showed significant upregulation in GDF15 protein expression in both skeletal and cardiac muscle tissue of SED+T mice, but not WR+T counterparts, suggesting a unique physical activity-mediated muscle protection in cancer cachexia. Therefore, our study supports the suggestion of GDF15 as a promising muscle wasting marker that may act as a biomarker for the early detection of cachexia and a potential therapeutic target in the clinical setting. Additionally, even small amounts of physical activity seem to impact the expression of these growth differentiation markers and may have the potential to protect against skeletal and cardiac muscle wasting in cancer cachexia. With the combined upregulation of inflammation, metabolic dysfunction, and muscle wasting in our study, further research should investigate whether GDF15 acts as a link between these upregulated pathways in cachectic cancer patients.

## 5. Conclusions

In the present study, we have identified the tumor-suppressive, myoprotective, and cardioprotective potential of even small amounts of physical activity concurrent with tumor burden. While daily physical activity levels steadily declined throughout the tumor-bearing period, a threshold of physical activity was maintained in the WR+T group. Therefore, even small volumes of voluntary wheel running protected skeletal and cardiac muscle from tumor-mediated dysfunction and atrophy. These encouraging preclinical findings provide a strong foundation for translational studies interrogating whether moderate voluntary exercise can be an effective nonpharmacological, protective measure against cancer-mediated muscle wasting.

## Data Availability

Data available upon request from the authors.
